# The Current Status of the Kenya Capsian

**DOI:** 10.1007/s10437-016-9211-5

**Published:** 2016-03-04

**Authors:** Alex Wilshaw

**Affiliations:** Leverhulme Centre for Human Evolutionary Studies, University of Cambridge, Cambridge, Cambridgeshire UK

**Keywords:** Later Stone Age, East African archaeology, Kenya Capsian, Eburran, Kenya, Aurignacian

## Abstract

East Africa is home to a rich array of stone-tool traditions that span human prehistory. It is unsurprising, therefore, that the region attracted pioneer prehistorians in the early twentieth century, including L. S. B. Leakey, E. J. Wayland and T. P. O’Brien, who created the first cultural framework for East African prehistory during the 1930s. Although aspects of this framework remain relevant today, others have become misunderstood relics of an old classification system that hinders current research. This is particularly evident in the classification of a Later Stone Age (LSA) culture – the Kenya (East African) Aurignacian, later known as Kenya (East African) Capsian. Although this cultural entity was redressed during the 1970s and 1980s and redefined as the Eburran industry, there is still mystique surrounding the current status of the Kenya Capsian, its original scope and definition, the relationship with the Eburran and its position within a modern understanding of the East African LSA. This is largely due to paradigmatic shifts in researcher attitudes, leading to the use of the Eburran as a false proxy. It is necessary now to completely remove the term *Kenya Capsian* as an indication of similarity among the different LSA technologies. However, there also needs to be less emphasis on the importance of the Eburran and recognition that it is just one example of a multitude of diverse localised LSA industries. This will open the way for future research into the LSA and facilitate our greater understanding of recent prehistory in East Africa.

## Résumé

L’Afrique de l’Est est le berceau d’un riche ensemble de traditions qui couvrent toute la préhistoire humaine. Il est donc peu surprenant que cette région ait attirée au début du vingtième siècle les pionniers de la préhistoire, dont L. S. B. Leakey, E. J. Wayland et T. P. O’Brien, qui établirent les premiers systèmes culturels de l’Est Africain dans les années 1930. Bien qu’un certain nombre d’aspects de ce système reste pertinent aujourd’hui, d’autres constituent aujourd’hui les reliquats mal interprétés d’un vieux système de classification qui entrave la recherche actuelle. Cela est particulièrement clair pour la classification de la culture de l’Age de Pierre Tardif (APT) – l’Aurignacien du Kenya (Est Africain), connu ensuite sous le nom de Capsien du Kenya (Est Africain). Bien que cette entité culturelle ait été révisée pendant les années 70 et 80, et redéfinie comme industrie de l’Eburrien, une certaine confusion entoure toujours le statut actuel du Capsien du Kenya, son ampleur originale et de sa définition, sa relation avec l’Eburrien, et sa position vis-à-vis de notre vision de l’APT de l’Est Africain. Cela s’explique en grande partie par les variations paradigmatiques des chercheurs, conduisant à utiliser abusivement le terme Eburrien. Il est désormais nécessaire de complètement abandonner l’expression Capsien du Kenya pour indiquer une similarité entre technologies de l’APT. Cependant, il est également nécessaire de moins insister sur l’importance de l’Eburrien, et de reconnaître qu’il s’agit juste d’un exemple parmi d’autres d’une multitude d’industries locales de l’APT. Cela devrait ouvrir la voie à de futures recherches sur l’APT et faciliter une meilleure compréhension de la préhistoire récente de l’Afrique de l’Est.

## Introduction

East Africa is awash with a diverse array of Later Stone Age (LSA) lithic assemblages that share common morphologies yet retain idiosyncratic features. The first reports of stone-tool evidence from the region were written at the end of the nineteenth century, as a result of which serious investigations into these lithic industries have been underway for nearly a century (Dewey and Hobley 1925; Gregory 1896; MacDonald 1923; Wayland 1924). Such an extensive research history has inevitably resulted in the use of a wide variety of localised archaeological nomenclature, which can be quite perplexing when viewed retrospectively. When multiple sets of terminology have been applied to the same archaeological evidence (be it sites, assemblages or tool traditions), it can be difficult to understand how the different terms equate, if at all, and even trickier to rectify the evidence within a single cohesive framework. This is especially relevant when dealing with museum collections that are catalogued and described under an antiquated classification system. Furthermore, understanding the status of an assemblage or site within a modern framework of nomenclature is essential to give it utility for contemporary study. These issues are exacerbated when the names or phases within different types of classification system do not correspond spatially or temporally. This is currently the case with what is commonly known as the Kenya Capsian, a Later Stone Age tradition of particular importance, as it immediately predates and overlaps with food producers.

This stone-tool tradition was defined by Louis Leakey in the 1920s and early 1930s (Leakey 1931). It began life as the Kenya Aurignacian in recognition of its general parallels with the European Upper Palaeolithic, at a time when the European sequence was the point of reference for prehistory. The scope of the “Kenya Aurignacian” was expanded geographically in the 1930s to become the East African Aurignacian (Leakey 1936). In the 1940s, Leakey replaced the *Aurignacian* component of the name with *Capsian*, after he recognised similarities with the Capsian of North Africa (Leakey 1947). The motivation for this change was likely the influence of colleagues from Cambridge and Africa (Cole 1975), who, like Leakey himself, recognised that there were terminological issues arising from the use of European nomenclature and recommended an African name for an African industry (Leakey 1950). Such recommendations would later be ratified by the Burg-Wartenstein protocol on precision and definition in archaeological terminology (Clark et al. 1966).

Further work by Cole (1954) refined the concept and popularised the name Kenya Capsian. In the 1980s, a reassessment of a proportion of the Kenya Capsian sites and artefacts was carried out; a new generation of researchers redefined this techno-complex and renamed it the Eburran industry (Ambrose 1984a, 1985, 1998; Ambrose et al. 1980). It is to the Eburran and its associated phases and definition that researchers continue to refer today (Kusimba 2013). However, the situation is not as straightforward as it at first may seem. The Eburran was not a direct replacement for the Kenya Capsian, and, as no alternative affinities were suggested for Kenya Capsian sites that were excluded from the new framework (other than that they “must now be referred to other industries” [Ambrose et al. 1980, p. 249]), it consequently neither fully removed the name nor satisfactorily explained the evidence previously attributed to the Kenya Capsian. To compound this, the characteristics used to define the Eburran do not correspond to those definitive of the Kenya Capsian nor are they exclusively lithic in nature (Ambrose 1984a, pp. 236–247). This makes it difficult to attribute sites that were not specifically listed or published as Eburran and that lack decisive ecological, spatial or temporal data (*ergo* those sites that comprised the Kenya Capsian). As a result, much miscomprehension surrounds the current status of the Kenya Capsian, its original scope and definition, the relationship with its purported terminological successor the Eburran and its position within a modern understanding of the East African LSA.

This miscomprehension is largely the unforeseen result of altering perceptions in archaeology, as well as of the original Kenya Capsian. The privilege of a long research history is often a double-edged sword, and the longer the history, the greater the potential complexity of theories, concepts, paradigms and methods that have been applied to any given topic. Understanding the historical context of, and intellectual influences on, researchers of the past is essential for understanding how their work should be perceived within a contemporary context. This reduces the risk of misinterpretation and misapplication of older research, not through intent, but simply because the researchers do not share a common intellectual time or space. The only way in which to elucidate is to return to the beginning and try to understand the evolutionary history of not only this specific industry, but also the changing paradigms amongst East African prehistorians.

This paper offers a review of the historical development of the Kenya Capsian, as defined by Leakey, and its ever-changing names – from the Kenya Aurignacian to the East African Aurignacian, the East African Capsian, the Kenya Capsian (the most renowned of the four earlier names) and eventually to the name by which it is, mistakenly, referred to today, the Eburran. This clarification aims at not only simplifying the situation for those endeavouring to work with older museum collections but also expounding the current situation of the Kenya Capsian as a terminological relic.

## The Kenya Aurignacian: the Birth of the Original LSA Classification System

The Kenya Aurignacian was the name given by Louis Leakey to a Late Pleistocene-Holocene, East African Mode 4-5 blade-based microlithic technology. The industry, amongst others, was outlined as part of a framework for Kenyan stone-tool technologies in *The Stone Age Cultures of Kenya Colony* (Leakey 1931). Although other researchers had been working in East Africa before this time, Leakey’s study was the first attempt at a coherent framework for prehistory in East Africa.

**Fig. 1:**
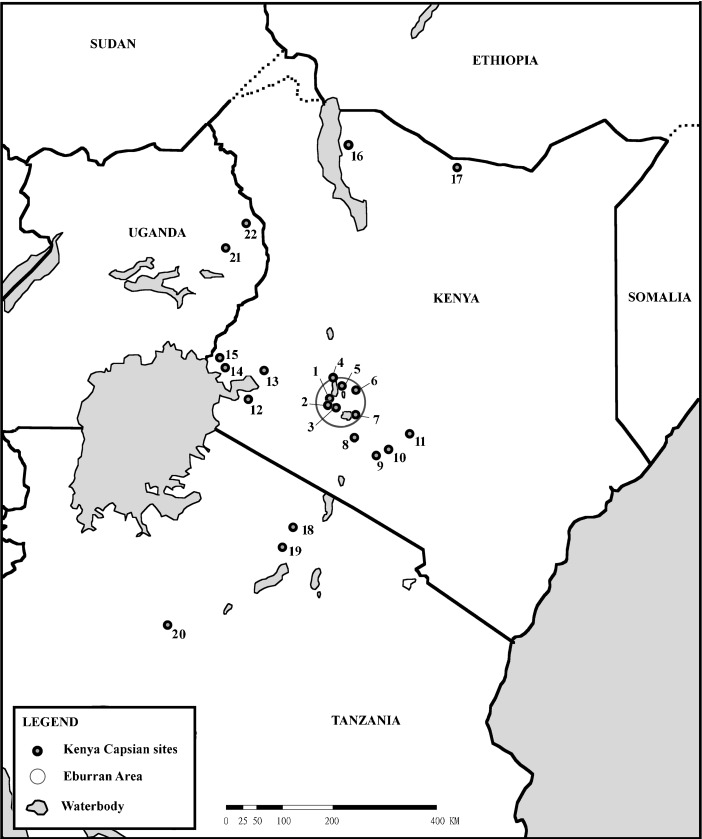
Kenya Capsian and Eburran ranges in Eastern Africa. Including a selection of sites: *1* Nderit Drift, *2* Gamble’s Cave II, *3* Prospect Farm, *4* Hyrax Hill, *5* Lion Hill Cave, *6* Cartwright’s Site, *7* Naivasha Railway Rockshelter, *8* Knightwick, *9* Kabete, *10* Thika, *11* St. Austin’s Mission, *12* Alara River and Sore, *13* KUR Line, *14* Ngiya churchyard and Usenge, *15* Ndenga, *16* Nderati, *17* Ele Bor, *18* Apis Rock, *19* Olduvai Gorge, *20* Ulanga Maru, *21* Napak, *22* Moroto. Tendaguru not shown on map

**Fig. 2:**
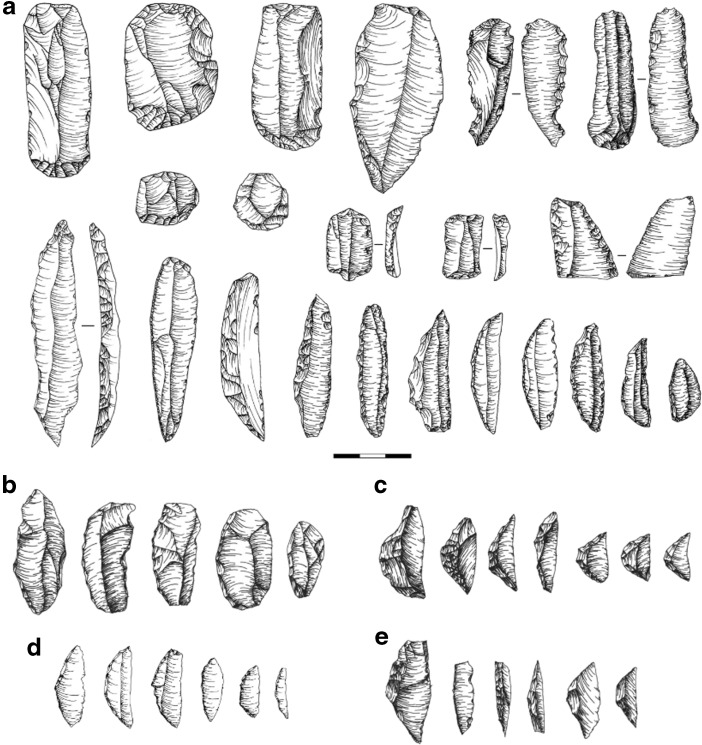
**a** Selection of obsidian stone tools of the Kenya Capsian, later Eburran, industries. Backed blades and crescents from **b** Q19, Ngiya churchyard, Nyanza, Kenya (basalt); **c** Q15, Alara River, Nyanza, Kenya (chert); **d** Q20, Apis Rock, Tanzania (chert and chalcedony); and **e** Q14, Ndenga, Nyanza, Kenya (chert). **a** after Ambrose (1984b, 1984a, 1985). All illustrations are by the author

The Kenya Aurignacian is characterised by large and abundant backed blades, geometric microliths and burins and common end scrapers. In its original formulation, the industry consisted of five phases: Basal Aurignacian, Lower Aurignacian and Upper Aurignacian A, B and C (Leakey 1931). The phases are extensionally defined, (rather than being intentionally defined, with a series of quantified attributes that characterise each phase), with type sites that represent the different phases and can be used to compare to novel evidence. The type sites consist of Cartwright Site (Fig. 1, 6), Nderit Drift (Fig. 1, 1) and Gamble’s Cave II (Fig. 1, 2), forming the type sites respectively for phases A, B and C. All of these type sites are found within the Nakuru-Naivasha Basin in central Kenya, where Leakey spent most of his childhood and early career working on his doctoral thesis and with the East African Archaeological Expedition (EAAE).

The material included within the Kenya Aurignacian industry is well known and has been well described for the type sites in the Nakuru-Naivasha Basin (Ambrose 1984a, 1985, 1986, 1998, 2002; Ambrose et al. 1980; Cole 1954, 1963; Hivernel 1974; Leakey 1931; Wilshaw 2012a). However, Leakey described three further geographical groupings as Kenya Aurignacian. These include sites in (a) the Central Province, (b) Nyanza Province and (c) Turkana (Fig. 1, 15–18), to which publications rarely refer and for which collections exist in Kenya National Museum with little or no documentation (Wilshaw 2012b). A brief provenance for each of these early collections, where known, is presented in Table 1. Each collection is labelled (for the most part) with a note card written in Leakey’s hand and using his letter:number:letter classification system (e.g., Q.1.a.). Despite the distribution of affine lithic scatters/assemblages across Kenya, it is evident that he considered all of these unpublished sites and artefacts as part of the Kenya Aurignacian tradition. The location of the Kenya Aurignacian type sites in the Nakuru-Naivasha Basin is likely a reflection of both the abundance and excellent condition of artefacts in the area and not because Leakey considered the industry to be confined to the Central Rift.

**Table 1:** Data related to early unpublished sites attributed to the Kenya Aurignacian from Kenya, Uganda and Tanzania

Area	Map no. and site	Year	Providence	Affinity	Artefact count	Leakey’s site ID	KNM no.
Central Kenya	9. Kabete	1928, 1932	Surface finds from the East African Archaeological Expedition (EAAE) and a donation from Charles Wainine	Upper Kenya Aurignacian	16, 6	Q.1.a, Q.1.a.1	596–597
	10. St. Austin’s Mission	1929	Surface find discovered by the EAAE near Nairobi	Upper Kenya Aurignacian C	21	Q.7	621
	11. Thika	1945^a^	Presented by R. H. Pullen Burry	Upper Kenya Aurignacian	108	Q.17	634
West Kenya	12. Alara River	1942^a^	Collected and presented by Archdeacon Owen	Kenya Aurignacian	29, 71	Q.13, Q.13.a	630–631
	12. Sore	1942^a^	Collected and presented by Archdeacon Owen, found on the 9.1-m (30-foot) beach of Lake Victoria	Upper Kenya Aurignacian	39	Q.10.a	627
	13. KUR Line	1938	Artefacts from multiple sites along the KUR Line, collected by M. D. Leakey and L. S. B. Leakey	Upper Kenya Aurignacian A	400	Q.4.a.2	604
	14. Ngiya Churchyard	1942^a^	Collected and presented by Archdeacon Owen. Found whilst excavating church foundations. See Owen (1939)	Kenya Aurignacian	15	Q.19	636
	14. Usenge	1942^a^	Collected and presented by Archdeacon Owen, found on a steep rocky hillside without stratification. See Owen (1939)	Kenya Aurignacian	5	Q.11	628
	15. Ndenga	1942^a^	Collected and presented by Archdeacon Owen	Late Kenya Aurignacian	20, 142	Q.14, Q.14.a	632–633
North Kenya	16. Nderati	~1930	Mention of Aurignacian at Nderati. Small excavation and collection was later made at Nderati Wells by Barthelme (1977)	Kenya Aurignacian	50	L.6	No data
	17. Ele Bor	~1920–1930	No data on early collections, but later works for Ele Bor A include Chittick (1976), Phillipson (1976) and Gifford-Gonzalez (2003)	Kenya Aurignacian	No data	No data	No data
Tanzania	18. Apis Rock	1931	Artefacts from section 7 of an excavation. References include Leakey (1936), Leakey et al. (1972) and Masao (1979).	Upper Kenya Aurignacian C	53	Q.20	642
	19. Olduvai Gorge	1931	Presented by L. S. B. Leakey from Bed V, Gamblian surface, contemporary with the human fossil labelled OH1 (Wayland 1932). References include Leakey (1979)	Upper Kenya Aurignacian C	31, 28, 268, 16, 36	Q.15., Q.15.a–d	637–641
	20. Ulanga Maru	ca. 1930	Found in a stream bed 15 miles east of Nzega, 18 km (11 miles) northwest of Zilza; presented to the museum by D. R. Grantham.	Kenya Aurignacian	5	J.95	346
	—. Tendaguru	1924	Surface artefacts collected by Leakey whilst excavating dinosaur fossils for the Natural History Museum (Cole 1975)	Kenya Aurignacian			348
Uganda	21. Napak	1923	In stratigraphy in gravels at Napak. References include Wayland (1924, 1934)	Kenya Aurignacian	No data, although O’Brien and Solomon (1939) refer to large collections from Karamoja *ca.* 1925		
	22. Moroto	1923	Artefacts from Moroto, Karamoja. References include Wayland (1924, 1934)	Kenya Aurignacian	No data, although O’Brien and Solomon (1939) refer to large collections from Karamoja *ca.* 1925		

^a^The date of donation, rather than the date of collection

**Table 2:** Description of main characteristics of the different phases of the Eburran, adapted from Leakey (1931), Cole (1954, 1963), Ambrose et al. 1980 and Ambrose (1984a, 1985, 1998)

Eburran phase		Date	Defining features	Kenya Capsian phase	Representative sites
Giant Blade Eburran	I	10.3 kya (but believed to extend into the Late Pleistocene ∼12 kya)	Mean microlith length >49 mm (crescents commonly being over 70 mm in length; the large size of these backed tools characterises this phase). Small scrapers (mean length ∼29 mm). Tool frequencies similar to those of phase II, but microlith, scraper and *outils écaillés* morphology differs (although the differences are not described)	No equivalent	Nderit Drift (section 25)
Large Blade Eburran	II	11.0–10.3 kya	Mean microlith length 33–36 mm. End scrapers are considerably larger than those of phase I. Fine retouch prepared platforms directed from the release face of the core onto the platform	Lower Kenya Capsian	Nderit Drift (section 13). Marula Rockshelter (talus slope, L3). Prospect Farm. Masai Gorge Rockshelter (stratum 2)
	III	(midpoint) 8.5–8 kya	Mean microlith length 32–37 mm. End scrapers are considerably larger than those of phase I	Upper Kenya Capsian A	Gamble’s Cave II (Leakey’s level 4, Nelson’s levels 1–12). GsJi29 Nderit Drift Hippo site. Marula Rockshelter (main occupation)
	IV	∼7.8–6.6 kya	Mean microlith length 26.2 mm. Geometric microliths, end, convex, notched and side scrapers decrease in frequency. Burins and nongeometric microliths increase in frequency. Inversely retouched flakes become more common. *Outils écaillés* reach a significant frequency	Upper Kenya Capsian B	Gamble’s Cave II (Leakey’s level 4, Nelson’s levels 13–25). Lion Hill Cave (lower occurrence). Salasun (strata V–VI). Enkapune Ya Muto (RBL3, DBS1, RBL2–3 and RBL2–2). Naivasha Railway Rockshelter (5–7)
Small Blade Eburran	Va	4.5–1.8 kya	Phase IV lithics with mean microlith length 20–25 mm accompanied by	C, D, Neolithic variants	Enkapune Ya Muto (RBL2–1, RBL1, BS1). Masai Gorge Rockshelter (stratum 3). Occupation level 3, layer 12 at Gamble’s Cave II (Leakey’s level 3, Nelson’s layer 12). Naivasha Railway Rockshelter (levels 1–4). Pickford’s Site
			• Pottery and domesticates		
			• Ecotonal environment		
			• Cave/shelter sites		
			Emphasis on obliquely truncated, rather than curved backed blades		
	Vb	4.5–1.8 kya	Phase IV lithics with mean microlith length 20–25 mm accompanied by:	C, D, Neolithic variants	Salasun (Strata II–IV). Hyrax Hill
			• Pottery and domesticates		
			• Grassland environment		
			• Open-air sites		
			Emphasis on obliquely truncated, rather than curved backed blades		

Ages represent calibrated radiocarbon dates measured in thousands of calendar years before the present (kya)

## The East African Aurignacian: the Growth of the Classification System

The original discoveries made by Leakey, the EAAE and missionary or administrative workers in Kenya firmly established the Kenya Aurignacian as both an entity in East African prehistory and a prehistoric classification system for some of the stone-tool variation observed (Leakey 1931). Naturally, as the evidence grew, so did the framework developed to incorporate it. Throughout the 1930s, references to the East African Aurignacian began to appear in place of the Kenya Aurignacian, and sites from both Tanzania and Uganda were subsumed into the classification system (Leakey 1936; O’Brien 1939; Wayland 1934). Recorded within the Kenya National Museum collections are four sites from Tanzania that were included within this East African framework and a further two concerning Ugandan sites referred to as such – the details of which are shown in Table 1.

It may be noted from the site descriptions in Table 1 that several of the Kenyan and non-Kenyan sites were assigned to the East African Aurignacian not by Leakey, but by other prominent researchers of the period. Some would argue that this casts doubt upon whether or not Leakey himself would have classified the sites in the same manner, and the extent to which researchers were qualified to recognise and attribute such artefacts to the East African Aurignacian. However, the experience and expertise of the researchers assessing the affinity of the Ugandan LSA traditions at the time – Archdeacon Owen, T. P. O’Brien and E. J. Wayland – cannot be called into question. All had first-hand exposure to the collections – Owen’s sites had been visited by both Leakey and Wayland (amongst other prominent names) (Owen 1938); O’Brien worked and commented often on collections from Kenya, Uganda and Tanzania (O’Brien and Solomon 1939); and although Wayland focused largely on the Ugandan record, he visited the Central Rift lakes in early 1927 and Olduvai Gorge in 1932, in order to assess the age of the stratigraphic sequences and their associated technologies at the request of Leakey. The two researchers evidently agreed that their observations on the regional LSA industries were similar, and Wayland (1934, p. 343) concurred with Leakey that the Olduvai collections were “Kenya Aurignacean.”

Wayland would later write that “the [East African] Aurignacean [*sic*] appears to have been a foreign influence which came, presumably, from the north or more likely the north-east for in that direction [East African] Aurignacean sites are commonest indeed, they would seem to be decidedly rare elsewhere in Uganda” (Wayland 1934, p. 351). The explicit statement that artefacts from the East African Aurignacian were rare in the interior of Uganda (echoed by O’Brien and Solomon [1939]) adds weight to the argument that the classification framework was indeed intentionally aimed at describing an East African phenomenon. There was clear selection of which sites and industries were and were not Aurignacian, and the broadening of the name from *Kenyan* to *East African* was *intentionally* done, not because researchers working outside of Kenya were abusing the existing classification as a “dumping ground” for non-Kenyan sites, but because they considered the features (mostly typological) of some LSA assemblages outside Kenya to justify the suggestion of a regional prehistoric culture.

The last reference to the East African Aurignacian appeared in 1939 (O’Brien and Solomon 1939), and the brevity of use is probably the result not of any conscious decision on the part of the researchers involved, but as an unforeseen result of the tumultuous times through which they lived. This is aptly reflected in the date of the penultimate publication to mention the East African Aurignacian – 1939. The outbreak of the Second World War threw the future of the colonies and protectorates into jeopardy. E. J. Wayland was posted back to England on war service and did not return to Uganda until his retirement in 1953 (Davies 1967). Likewise, T. P. O’Brien served out much of the war in India, with the eventual intention of returning to Uganda to carry out further fieldwork in the 1960s; unfortunately, he died in 1968, before this could be realised (Posnansky 1968).

## The East African Capsian: an African Name for an African Industry

Unlike Wayland and O’Brien, Leakey stayed on in Kenya throughout the war as part of the African Intelligence Service (Leakey 1974). He continued, when he could, to research a diversity of prehistoric periods, although publications were limited by the war. When he finally published again on the East African Aurignacian, it was to replace *Aurignacian* with *Capsian*. However, he made it clear that *Capsian* was very much a term for “our East African blade and burin culture”, and not just a Kenyan phenomenon (Leakey 1947, p. 206). This would be his final dedicated publication on the topic.

As a result of the domineering role of individual characters in the early days of prehistoric research in Africa, the loss of engagement from these three pioneers had a profound effect on research into the LSA of the area, as well as the manner in which the earlier research was understood. It is likely that the initial emphasis on Kenyan sites and the poorly established use of the term *East African* led to the assumption that Kenya (and in particular the central Kenyan Rift Valley) was the spatial extent of the local Capsian tradition. However, this is not the case nor was it Leakey’s intention that the industry should be understood within such a confined geographic area.

Leakey, along with his contemporaries, did not think in terms of localised industries, but rather took a macro approach to stone-tool technologies on a regional or continental scale. These pioneer researchers did not have the privilege of the knowledge that we have today. For example, the gains that have been made in population genetics, which highlight the importance of localised patterns and processes on the archaeological record, or access to high-resolution spatial data, climatic reconstructions or chronometric dating methods aids in the construction and interpretation of our understanding of prehistory in a way that was inaccessible to Leakey and others. At the time, very broad pictures of prehistory were still being built. Prehistoric archaeology was a relatively new discipline and, although it had been developing in Europe for over a century (Boucher de Perthes 1847; D’Acy 1875; Evans 1860, 1872; Frere 1800; Schmider 1850), the African prehistoric record was largely unknown. Therefore, when Leakey undertook to create a framework for the stone tools he had observed in Kenya, he naturally used and applied the classificatory systems to which he had been exposed in the more developed field of European prehistoric archaeology, something clearly reflected in his initial use of the “Aurignacian” nomenclature (Sutton 1974) and in his application of the industry across a broad geographic area. An understanding of both Leakey’s intentions for this industry and the context within which it was developed is essential for understanding the current status of what would shortly, misguidedly, be named as the Kenya Capsian industry.

## The Kenya Capsian: Consolidation and the Causation of Misapprehension

Following the war, research into the East Africa Capsian was not really carried out again until the seminal work of Cole (1954) on the prehistory of East Africa, in which she expands the industry to include two further phases: the Upper Kenya Capsian D and the Neolithic variants, with the respective type sites of Naivasha Railway Rockshelter and Hyrax Hill (Cole 1954, 1963). It should also be noted that Cole prefers the term *Kenya* Capsian, rather than referring to Leakey’s *East African* Capsian, despite recognising that the industry existed in both Kenya and Tanzania (Cole 1954) (the few sites in Uganda having already been disassociated with the Capsian [O’Brien 1936, 1939]).

The poor establishment of the term *East African Capsian* from Leakey’s ultimate dedicated publication on the topic (Leakey 1947), and the success of Cole’s article (and later book) promoting the use of the term *Kenya Capsian*, resulted in a sweep of the earlier terminology and the loss of recognition in the nomenclature that the Capsian extended to sites outside of Kenya. The Kenya Capsian is invariably attributed to Leakey and, although he did use the term in occasional publications prior to this time (Leakey 1951), it is not until after Cole’s publication that L. S. B. Leakey himself begins to use the term consistently. It is somewhat ironic, one feels, that he initially does so in relation to artefacts from Tanzania (Leakey 1968). Likewise, M. D. Leakey continues this practice after her husband’s death, and, although recognising and describing differences between the Kenyan Capsian and an industry from Olduvai Gorge (and another one from Apis Rock, Tanzania), she reinforces the switch in nomenclature by suggesting that the Tanzanian industries were a “local variant of the Kenya Capsian” (Leakey et al. 1972, p. 340).

It is at this point that the cracks in the Capsian framework begin to appear and there is a divergence from evidential reality. The introduction of the term *Kenya Capsian* applied continuously to non-Kenyan sites creates an obvious paradox that must have been evident even in the initial publication of the name. It is unclear how this came to pass, but it may be a reflection of the convergence of two different generations of researchers, neither of which fully understood the others’ paradigmatic outlook and intentions for the framework, and an abundance of evidence that could no longer be explained within the bounds of the Capsian. A complete revision of the classification system was, in retrospect, necessary by the early 1960s, but redress would take another decade for consideration and be another two decades in the making.

## The Eburran: a False Proxy; Not Just Another New Name but a New Industrial Concept

By the end of the 1970s, it was apparent that what was now known as the Kenya Capsian classification system, developed under a different name by L. S. B. Leakey and added to by others, could no longer cope with the diversity exhibited in the East African LSA. The expansion of research projects across East Africa during this time revealed an unprecedented complexity of diverse and localised traditions within the LSA (Bishop and Posnansky 1960; Bower et al. 1977; Gramly 1976; Nelson 1973; Robbins 1974), which neither the Kenya Capsian, nor indeed any such geographically broad classification system, could feasibly represent. Archaeological understanding had increased, and a paradigmatic shift towards greater contextualisation of prehistoric cultures within their palaeoenvironments, as well as better analytical techniques, led to a reassessment of the industry.

The aims of the researchers had also changed by this time, and, unlike Leakey, they took a microevolutionary approach to studying LSA industries: not broadly within East Africa as a whole, but specifically within localised areas. Following these changes, a novel technological framework was introduced which redefined only part of the Kenya Capsian as the Eburran – one of several local variants of the LSA that were becoming increasingly apparent and had previously been subsumed within the Kenya Capsian. The Eburran initially consisted of phases I–IV (Ambrose et al. 1980), with later amendments to merge phases II and III and add a phase V (Ambrose 1984a, 1985). The Eburran moved away from extensional definitions and towards the use of tangible characteristics, which are laid out in Table 2, with basic illustrations in Fig. 2.

The phases of the Eburran industry correspond, to an extent, to those of the Kenya Capsian. However, Ambrose was very clear that not all sites included in a Kenya Capsian phase were included in its equivalent Eburran phase and that only technologies from very specific Kenya Capsian sites were representative of the new Eburran phases (also detailed in Table 2) – specifically those from the Nakuru-Naivasha Basin in Kenya.

The description of the Eburran as a local tradition represented an improved LSA chronology for the area, as well as a basic quantitative description of the technology and new hypotheses about the relationship between the industry, its makers and the ecological niche that they inhabited (Ambrose et al. 1980; Ambrose 1985; Bower et al. 1977). The Eburran, as conceived, was radiocarbon-dated between 12,710 ± 310 bp and 1,110 ± 115 bp (Ambrose 1980; Ambrose 1984a, 1984b; Bower et al. 1977). Simultaneously, research into the changing climate and ecology of the Late-Pleistocene/Holocene Nakuru-Naivasha Basin was undertaken, which allowed the newly dated archaeological record to be contextualised within an environmental framework. This led to the development of a key hypothesis related to the adaptations of its makers.

The Late-Pleistocene/Holocene period was characterised by wet and dry phases (pluvials and interpluvials) that caused a series of associated changes in lake levels; these changes ranged from full lake overflow (when the lakes of Nakuru and Elmenteita formed a single body of water) to complete desiccation, which resulted in an archaeological hiatus between 6.0 and 4.0 thousand years ago (kya). Within the lake basins, there are three different types of ecologies that exist in bands at different altitudinal levels – savannah (lowland), bush-woodland (mid-range) and montane-forest (highland). The climatic changes caused these ecological bands to migrate altitudinally; wet phases led to the expansion of bush and montane-forest into lowland areas, and arid phases saw the expansion of savannah lands to higher altitudes.

Ambrose et al. (1980) argue that the makers of the Eburran industry primarily exploited the band of savannah-montane forest ecotonal areas. They identify altitude as a potential adaptation of the makers to a specific ecotonal niche, *ergo* the ecology and marginal environments that are associated with different altitudes at different points in prehistory, to be a factor in the appearance and evolution of the industry. Essentially, the model proposes that the location of Eburran sites, and the intensity of their occupation, migrate over time with the migration of the ecological niche to different altitudinal levels in response to the climatic changes (Ambrose 1986; Ambrose et al. 1980). Later research also suggested that the Eburran had two different adaptations – one represented by the use of cave sites at higher ecotonal altitudes and the other by the use of open-air savannah sites at lower altitudes, after 4.9 kya. It further hypothesised that the latter was associated with a gradual shift towards animal domestication in the later stages of the Eburran in response to the spread of the Pastoral Neolithic (Ambrose 1998; Kusimba and Kusimba 2005; Lane 2011; Mutundu 2010).

The introduction of the ecotonal adaptation model had three consequences for the interpretation of LSA archaeological sites in and around the Nakuru-Naivasha Basin. Firstly, the use of a suite of defining attributes, including lithic technology, ecology and the presence of both pottery types and domesticates, clearly made inferences about the Eburran as a techno-complex that had not been inherent within the Kenya Capsian. Secondly, the novel classification excluded some sites previously identified as Kenya Capsian from belonging to the Eburran industry, particularly those from non-ecotonal areas. Ambrose et al. (1980, p. 249) stated that “many of the [Kenya Capsian] surface collections … must now be referred to other industries”, although they do not suggest which. Phillipson (2005, p. 126) would later refer to these sites as “generally ubiquitous”, and that “no really convincing and meaningful classification of them has yet been proposed”: evidence everywhere, which was accounted for nowhere. Thirdly, after extensive survey work (Ambrose 1984a, 1985; Ambrose et al. 1980; Bower et al. 1977), the original geographic range of the Kenya Capsian industry was redefined following the exclusion of many smaller occurrences. Dealing with off-site archaeology and surface scatters is difficult within the Eburran techno-complex – largely because frequency variables are invalid due to the small unrepresentative sample sizes and contextual data required to confirm ecology, pottery or domesticates are absent or limited – and it is these types of sites that have since suffered the most from the introduction of the Eburran. The industry is stipulated to exist only within a 25 km radius of Mount Eburru, the extent of which is shown in Fig. 1 (Ambrose et al. 1980). Ambrose (1984a) later acknowledged that this geographical limitation may be too stringent, although a clarification/retraction was never issued.

The previous sections outlined the nomenclature, context, definition and attributes for the stone-tool culture that was popularised under the name Kenya Capsian, as well as for the Eburran classification that supplanted it. In the minds of many, the Kenya Capsian exists only as a historical classification system that has since been replaced, rather than a different conception of a prehistoric tradition, and it is this misconception that creates confusion.

As can clearly be established from the outline of the Eburran industry and its comparison to the Kenya Capsian (Table 2), Ambrose and colleagues’ proposal to rename and redefine the Kenya Capsian with the Eburran was not just a simple nomenclature change, replacing one name with another. What these studies did was to identify and define a single industrial entity, one strand of many that were encapsulated within the Kenya Capsian name, and to recognise it as an industry in its own right, different from other tool technologies that had previously been included *ensemble* under the Kenya (East African) Capsian umbrella. The situation is further exacerbated because the entity that was chosen as the basis for the Eburran definition had previously formed the original core of the Kenya Capsian: the obsidian industries of the Nakuru-Naivasha Basin, which include all of the type sites for the phases of the Kenya Capsian.

This has resulted in a situation where some researchers either see the two classification systems as directly analogous and misunderstand or misrepresent the relationship between them, their phases and associated definitions (Chmielewski 1987, p. 5; Clark 1988, p. 271; Hassan 2006, p. 79; Hirbo 2011, p. 18) or describe the Eburran as the local area’s exclusive LSA industry at the cost of “unassigned” sites which consequently remain unaddressed (Barham and Mitchell 2008, p. 327). In some cases it is possible to identify a corresponding Eburran phase for a Kenya Capsian site – particularly for those that lie within the Nakuru-Naivasha Basin, but the reality is that the majority of Kenya Capsian sites do not equate with a phase within the Eburran. The two industries are not comparable, as the Eburran industry is only a portion of the Kenya Capsian. In essence, Ambrose and colleagues recognised that Leakey’s classification system could not encapsulate the variation observed in LSA stone tools across East Africa, but instead of addressing and redefining the Kenya Capsian as a whole, they dealt only with a small portion of it. The way in which these two generations of researchers viewed their respective industries and how they thought about an East African framework for the LSA was different, Leakey taking a macro view of East Africa as a whole, while later researchers, with greater knowledge of LSA diversity, chose a micro approach that interpreted such variation in small-scale localised population processes.

In reality, there are many different localised cultural/ecological facies contained within the Kenya (East African) Capsian, encapsulated not only by those assemblages in the Nakuru-Naivasha Basin left out of the Eburran classification system (Ambrose et al. 1980) but also by the ones that exist in other geographic areas and have never been analysed for formal inclusion or exclusion from the Eburran system. As seen in Fig. 2, it is evident solely from the most abundant tool types – backed blades and geometrics – that the morphology and manufacture of the artefacts differ greatly across Leakey’s East African Capsian. The blades shown in Fig. 2a, which illustrates the typical Kenya Capsian tools, do form a coherent grouping, and the sites exhibiting such artefactual variation have been reclassified as the Eburran. But the lithics from the other four examples of assemblages (Fig. 2b–e) clearly exhibit a diverse range of morphologies. The lithics in the western Kenya example (Fig. 2b) have a far higher length-breadth ratio. They are also thinner, with rounding on the proximal and distal ends, even when compared to other western Kenyan variants (Fig. 2c). The latter are thicker, with more invasive backing retouch that is of greater comparability to the artefacts described by Robbins (1974) from Lothagam, than to the classic Eburran artefacts of the Central Rift. The microliths from Apis Rock, Tanzania (Fig. 2d) are probably the most similar to the Eburran and the Kenya component of the Kenya Capsian, which explains why Leakey continued to emphasise the inclusion of these collections within the *Kenya* Capsian. The pointed morphologies and slim nature of the Ndenga (Fig. 2e) artefacts are rare across all of these examples.

These are all reflections of the geographic and technological diversity encapsulated by the Kenya Capsian industry as originally formulated and described. They are not included within the Eburran reclassification and have no comparative Eburran phase, nor should they. Yet these are the materials most at risk of being incorrectly attributed to a phase of the Eburran industry. What they do represent are a selection of localised “Eburranesque” LSA industries that litter East Africa. Preliminary analyses of the collections held at Kenya National Museum suggest that there are examples of at least six such industries exhibiting a different range of variation (including the Eburran), and all of which fall within Leakey’s original East African Capsian (northern and southern Tanzania, northeastern Kenya, western Turkana and two from western Kenya), as well as those left orphan from within the Nakuru-Naivasha Basin.

So how should these industries be interpreted and what does this mean for the Kenya Capsian? Given that the lithic assemblages mentioned above have never been recategorised or included in the Eburran, their attribution continues to be to the Kenyan or East African Capsian. However, the Kenya Capsian is no longer meaningful in terms of how we understand the LSA of East Africa today. Such a conglomerate industry hides the diversity shown among these sites, restricting the comparative, contextual and in-depth analysis necessary to gain a greater understanding of LSA diversity between different cultural entities. Ultimately, given our greater understanding of the processes that underlie LSA diversity in East Africa, the Kenya Capsian can no longer be applied usefully, but only within a historical context.

The modern-day evidence for localised ethnic, biological and cultural diversity across East Africa is argued to be only a fraction of the diversity in prehistory (Lahr and Foley 1994). The population patterns, mechanisms and events that affect modern populations – migration, isolation, marginalisation, climate change and resource dearth – would equally have affected the East African stone-tool makers (Ambrose 1984a; Bower 1984; Gifford-Gonzalez 1998; Marlowe 2005). However, the exposure of different groups to such processes would not have been uniform (Olaka et al. 2010), especially given that the period was climatically variable across time and space, with evidence for highly localised environmental changes (Trauth et al. 2010). Much of the observed diversity in stone-tool cultures is likely to be the result of these localised population processes, and, therefore, it is understandable that a region-wide system of classification with a temporal depth of over 10,000 years, such as the Kenya Capsian, is neither representative of the local patterns observed nor adequate to cope with the level of cultural diversity produced as a result (Wilshaw 2012a). This is especially poignant given the paucity of our spatial and temporal resolution, in contrast to the incredible variation that would be expected.

In essence, all Kenya Capsian sites will *always* historically be Kenya Capsian sites, although they may not be Kenyan, because of the way in which Louis Leakey defined and understood the framework of the industry as comparable to the Aurignacian of Europe. However, it is necessary now to completely remove the term as an indication of similarity among the different LSA technologies in eastern Africa. It is likely that had the East African Capsian not been replaced by Cole with the popularised term *Kenya Capsian*, this issue would never have arisen. Any reference to an East Africa-wide industry of such variation would have been quickly removed in its totality by the end of the 1970s, and research may have been carried out across the range of LSA industries once encapsulated within the East African Capsian. As it stands, the vision of the Capsian as a Kenyan classification persisted and led to a situation where researchers saw the industry as geographically confined and as wholly replaced with the Eburran industry; the problem of LSA classification was therefore seen as largely solved. Ultimately, this has left many unexplored areas of the East African LSA, and sites such as those in Karamoja, Uganda, or the lesser known sites in Tanzania to remain in limbo, alongside the Kenya Capsian sites excluded from the Eburran industry both within and external to the Nakuru-Naivasha Basin.

It is also necessary to emphasise that just as the Kenya Capsian is no longer a useful entity, nor is the broad application of the Eburran classification system. Because the Eburran has received such detailed study and description by Ambrose and others, the industry is party to special treatment, referred to in books and articles regularly and discussed as a significant part of the East African LSA (Kusimba 2013, p. 464). Although the Eburran *is* special because of the density of sites and preservation quality of artefacts within the Nakuru-Naivasha Basin, it should be given no greater status than any of the other localised industries that were previously encapsulated in the Kenya Capsian. Instead, researchers need to view and discuss LSA diversity not as “the Eburran in Kenya,” but with recognition that there are many small-scale LSA variants in Kenya and elsewhere that are excluded from the Eburran and remain largely nameless and unrecognised.

Given the variety of smaller-scale population processes that created the diversity that we see today in the archaeological record, it is necessary for each “industry” or variant to be considered with far more equality than is presently the case. Only in this way can a realistic assessment of the number of contemporaneous traditions be made – whether they are LSA, Upper Palaeolithic, East African, South African or European. The variety of stone-tool technologies that characterises our later prehistory have the potential to reveal the social, economic and ecological strategies of hunter-gatherer communities after the environmental amelioration that took place in the Late Pleistocene and Early Holocene, and they should not be treated or interpreted as a single entity. This case study has focused on the Kenya Capsian and Eburran of East Africa, but doubtless similar arguments could be made for the geographically and temporally vast Aurignacian of Europe, which must mask myriad localised variations and with them the key to understanding the cause of the diversity observed. This is becoming increasingly relevant with the influx of ancient DNA studies revealing population structures and complexities beyond what was initially understood, explanations for which can only truly be gained from a greater understanding and resolution in the archaeological record. It is hoped, therefore, that this article will serve not only to correct and elucidate the understanding of the history of the LSA in East Africa and the status of both the Kenyan/East African Capsian and Eburran industries, that continues to vex researchers today, but also to set in motion a reassessment of other systems of classification that may be hindering the understanding and interpretation of recent human populations.
